# Nasopharynx cancer in Chinese of California.

**DOI:** 10.1038/bjc.1965.54

**Published:** 1965-09

**Authors:** P. Buell


BRITISH JOURNAL OF CANCER

VOL. XIX SEPTEMBER, 1965 NO. 3

### NASOPHARYNX CANCER IN CHINESE OF CALIFORNIA

P. BUELL

*From the Bureau of Chronic Diseases, California State Department of Public Health, Berkeley, California, U.S.A.*

Received for publication January 11, 1965

THE Chinese have an unusual predisposition for cancer of the nasopharynx whether they reside in Mainland China and the nearby islands of Formosa and Hong Kong (Hu and Yang, 1959; Yeh and Cowdry, 1954; Digby *et al.* 1941) or in more distant lands that have seen large settlements of Chinese, such as Indonesia, Singapore, New York City, and California (Djojopranoto and Marchetta, 1959; Muir, 1962; Martin and Quan, 1951; Zippen *et al.*, 1962). In most populations nasopharynx cancer is quite rare, accounting for probably less than 1 per cent of all malignant tumors. From what has been reported, the frequency in the Chinese seems to be greater by a factor between 10 and 50. When one considers also the relatively early age at which this malignancy occurs in the Chinese (Muir, 1962), it indicates, surely, an endemic condition of some importance.

Outside of Asia and its archipelagoes the largest number of Chinese are in California where they have dwelt for over one hundred years. They came in increasingly large numbers from 1854 until 1882 when the Exclusion Act prohibited entry to all but those in a few categories. Many of them were single or unattached family men, who came as sojourners intending to return eventually to the homeland, to enjoy what wealth they had sent back during the years of exile. And some did return; the Chinese population in California actually decreased during each decennial census from 1890 through 1920. By 1960 the population was about 95,000 and the number of families began to redress the imbalance created by single immigrant men.

The role of sojourner required that the traditional ways of life be maintained and this the Chinese probably did for a longer time than any other immigrant group. Even some of the second generation went to China for brides. If they are of the third, fourth and fifth generations, their cultural setting, according to Lee (1960), is more American than Chinese. Exceptions to this are not infrequent in San Francisco's Chinatown (Lee, 1960), which has not lost all the features of a ghetto, even though one of its principal supports is the tourist trade. Some 20,000 or more still reside in Chinatown.

In the data reported by Zippen and his colleagues (1962) on the Chinese in California, there was a suggestion of a reduced risk of nasopharynx cancer among the Chinese born in the United States. The data, limited to males, consisted of newly diagnosed cases reported to the California Tumor Registry, which covered some unknown proportion, but not all, of the Chinese cases in the state during the period 1942–57. There was, then, some lack of comparability between the coverage of cases and the enumeration of the population at risk.

The California mortality records, reported on below, have the disadvantage of understating the incidence of new cases of nasopharynx cancer, but probably not to a serious degree. Some advantage comes in having comparability between the coverage of deaths and of populations, and in having enough records for the period 1949–62 to study further the difference reported by Zippen *et al.* between the foreign and U.S. born, and to include the Chinese women.

### RESULTS

### *Nasopharynx cancer in the Chinese*

During the 14 years from 1949 through 1962 there were 67 deaths from cancer of the nasopharynx among Chinese men and 13 among the women. The Chinese population in California was enumerated in the Censuses of 1950 and 1960. Table I compares the average annual death rates for Chinese males during the first half and the second half of the 14 year period (based on interpolations for intercensal population estimates under the assumption of an exponential population growth). Similar average annual rates for Caucasoid males for the periods 1949–51 and 1959–61 are also shown in Table I.

TABLE I.—*Number of Deaths from Nasopharynx Cancer and Average Annual Rates per* 100,000 *Chinese and Caucasoid Males in California During Two Periods*

| | Chinese males | | | | Caucasoid males | | | |
|---|---|---|---|---|---|---|---|---|
| | 1949–55 | | 1956–62 | | 1949–51 | | 1959–61 | |
| Age | Deaths | Rate | Deaths | Rate | Deaths | Rate | Deaths | Rate |
| 15– . | 0 | — | 2 | 5·1 | 0 | — | 2 | 0·07 |
| 25– . | 3 | 6·2 | 5 | 8·2 | 0 | — | 3 | 0·10 |
| 35– . | 4 | 9·2 | 10 | 18·4 | 4 | 0·18 | 9 | 0·29 |
| 45– . | 11 | 29·9 | 12 | 27·6 | 16 | 0·88 | 18 | 0·72 |
| 55– . | 7 | 29·2 | 9 | 27·5 | 14 | 1·01 | 20 | 1·13 |
| 65– . | 3 | 27·1 | 1 | 5·9 | 10 | 1·20 | 27 | 2·29 |
| 75– . | 0 | — | 0 | — | 6 | 1·76 | 7 | 1·27 |
| Total, 15 & over | 28 | 13·4 | 39 | 15·4 | 50 | 0·45 | 86 | 0·57 |

Some increases in the rates appear in the youngest age groups, both for Chinese and White, but there is no systematic increase in each age group. It seems reasonable to combine all years, by getting weighted averages of the rates for the two periods in order to compensate for small numbers in each age group. These average annual rates over the whole period are graphed on semi-logarithmic scale in Fig. 1.

The 10-year age groups used for graphing are too broad for a definitive study of the age distribution of rates; and, as Fig. 1 reveals, a straight line may not really fit the data even up to age 55 in the Chinese. But a rough indication is given that up to about age 55 the Chinese males have better than a 40-fold greater risk of mortality from nasopharynx cancer than white males; after that age the difference appears reduced, though the data may be less trustworthy. The figure also reveals that not until about age 70 do the rates for Caucasoid males get as high as they are at age 20 for Chinese males.

The differences between the locally born and the foreign born Chinese are examined by computing the numbers of deaths each group would expect if they had the same age specific rates of mortality from nasopharynx cancer as the Caucasoid population. The ratio of the observed to the expected numbers of

FIG. 1.—Average annual death rates from nasopharynx cancer in Chinese (1949–62) and Caucasoid (1949–51, 1959–61) males, California. (Semi-log scale.)

deaths shows by what factor the risk is increased over that in the Caucasoid population of each sex.

The results in Table II show that the risk of nasopharynx cancer in the locally born Chinese is considerably higher than that in the white population, but lower than that in the immigrant Chinese. The factor of increase is about 20-fold for both the men and women of Chinese descent born in the United States, and 30 to 40-fold for the men and women born in China. Some reduction of risk seems to have taken place between the earlier and later periods in the locally born Chinese males.

TABLE II.—*Observed Numbers of Deaths from Nasopharynx Cancer Among the Chinese in California, and the Expected Number of Deaths if they had Experienced the Rates for the Caucasoid Population*

| | Chinese males | | | Chinese Females | | |
|---|---|---|---|---|---|---|
| | Observed deaths | Expected[1] deaths | Increase factor | Observed deaths | Expected[2] deaths | Increase factor |
| Both periods | | | | | | |
| U.S. born | 15 | 0·72 | ×21 | 3 | 0·15 | ×20 |
| Foreign born | 52 | 1·51 | ×34 | 10 | 0·23 | ×43 |
| First period, 1949–55 | | | | | | |
| U.S. born | 8 | 0·29 | ×28 | | | |
| Foreign born | 20 | 0·57 | ×35 | | | |
| Second period, 1956–62 | | | | | | |
| U.S. born | 7 | 0·43 | ×16 | | | |
| Foreign born | 32 | 0·94 | ×34 | | | |

[1] Based on rates by 10-year age group for Caucasoid males, California, 1949–51 and 1959–61.
[2] Based on rates by 10-year age group for Caucasoid females, California, 1949–51 and 1959–61.

The numbers of deaths entering into the differences between foreign and U.S. born are small, especially for the women. The difference for the men would be significant, in the conventional sense, at the 5 per cent level ; but a statistical significance test seems hardly appropriate. It took 14 years to collect the cases used and it is unrealistic to consider these cases as one of a number of samples of a population that would stay the same over a longer period of years.

The male/female ratios of nasopharynx cancer, when standardized for age differences (found to be necessary especially for the Chinese) are remarkably close : about 3·3 to 1 in the Caucasoid population and about 3·5 to 1 in the Chinese of California. This compares with a sex ratio of about 2·3 to 1 in data reported by Muir (1962) on the population of Singapore composed of about 75 per cent Chinese, 14 per cent Malaysian, 9 per cent Indian and Pakistani and 2 per cent others ; and 3 to 1 reported from China (Liang, 1964).

The only rates per unit of population that can be found from other lands are the aforementioned reported by C. S. Muir for the whole population of Singapore. Muir gives the age standardized death rate, computed by the method of Stocks (1959) for nasopharynx cancer in Singapore males as 4·5 per 100,000. For the California Chinese males the age standardized rate, also computed by the method of Stocks, is 10·0 per 100,000. If the Chinese of Singapore had all of the nasopharynx cancer, while constituting only 75 per cent of the population, their rate would still be considerably less than the rate for California Chinese males.

Muir gives figures indicating that the number of cancer deaths in Singapore coded to " pharynx, unspecified " (I.C.D. 148) amounted to 74 per cent of the nasopharynx cancer deaths, and many of them must be primary to the nasopharynx. For California Chinese these deaths come to about 19 per cent of the nasopharynx cancer deaths during 1949–62. Even so, when allowance is made for this difference of specificity in diagnosis and recording of pharynx cancer deaths, it is still fair to say that the immigrants to California have carried with them as much, if not more, risk of nasopharynx cancer as the immigrants to Singapore.

*Other groups in California*

Several other races and ethnic groups can be identified in the mortality records and census enumerations for California. The Negro population shows no unusual risk of nasopharynx cancer mortality: the males had 4 deaths in the period 1959–61 with 3·61 expected according to white rates; females had no nasopharynx cancer deaths and 1·45 expected. Immigrants from Mexico had during 1959–61: males 2 deaths with 2·22 expected, and females 1 death with less than one expected. On the other hand, the males of both the Japanese and Filipino populations in California revealed interesting differences: Japanese males, 1949–62, 7 observed nasopharynx cancer deaths and 2·80 expected; Filipino males, 1956–62, 6 observed with 1·32 expected. The Japanese and Filipino women had no observed deaths, but expectation was less than 1·0.

*Other cancer*

The mortality of the Chinese men from certain cancers of the head and neck during 1956–62 is shown in Table III. The numbers of expected deaths for each of the distinguishable parts of the pharynx are similar: oral mesopharynx 1·99; hypopharynx 1·13; nasopharynx 1·37. Yet, only for nasopharynx is there an excess incidence.

TABLE III.—*Observed Deaths Due to Selected Sites of Head and Neck Cancer in Chinese Males of California, 1956–62; Expected Deaths Based on Age-specific Rates for Caucasoid Males of California*

| Site | Observed number | Expected number |
|---|---|---|
| Tongue (141) | 1 | 4·47 |
| Salivary glands (142) | 3 | 1·08 |
| Mouth (143–4) | 4 | 3·77 |
| Oral mesopharynx (145) | 3 | 1·99 |
| Hypopharynx (147) | 1 | 1·13 |
| Nose, nasal cavities, etc. (160) | 4 | 1·13 |
| Larynx (161) | 5 | 7·16 |
| | 21 | 20·73 |
| Pharynx, unspecified (148) | 7 | 3·01 |
| Nasopharynx (146) | 39 | 1·37 |

There were 4 deaths attributed to primary cancer of the nose, nasal cavities and accessory sinuses, with 1·13 expected. This otherwise minor observation is mentioned because Yeh and Cowdry (1954) report an unusual frequency of cancer of the nasal and accessory cavities in a series of biopsy and autopsy specimens in Formosa; and also because the roof and lateral walls of the nasopharynx are covered with ciliated columnar epithelium continuous with that of the nasal cavity, while the rest of the pharynx is covered by stratified squamous epithelium.

Cancer deaths assigned to unspecified parts of the pharynx are set out separately in Table III because some of them may be nasopharynx cancers. Indeed, 6 of the 7 observed deaths in Chinese were described on the certificates simply as " lymphoepithelioma ", a common histopathological description of malignancy in the nasopharynx, and coding routine placed them in " pharynx, unspecified ". The result, with 7 observed and 3·01 expected deaths for Chinese men, suggests that failure to specify the part of the pharynx in the cancer mortality records of the white males cannot explain the remarkable difference in nasopharynx cancer experience between Chinese and white. Nor are the results, shown earlier, for Filipino, Japanese, Mexican and Negro, seriously affected by the classification " pharynx, unspecified ".

*Economic level*

Almost all of the death certificates for 1956–62 contain an entry reporting last occupation of the decedent which may be taken as a rough indication of economic level. Because Census returns report the occupational level only for the employed Chinese population, while the employment status before death cannot be determined, only a rough relative measure of the risk of nasopharynx cancer can be calculated.

Grouping at one level all owners and proprietors, plus professional, technical, sales, clerical and skilled workers, and at the other level all semi-skilled, service workers including cooks and laundrymen, and all laborers and farm laborers, the resulting age adjusted " rates " suggest about a 2-fold excess risk in the poorer class. If the unemployed and those out of the labor force at each age group are all assigned to the poorer class, a rather extreme assumption, the relative risk is about 1·5-fold. The apparent association with economic level might be due to sampling fluctuations, but it is worth noting.

It is not, however, open to a simple interpretation. Some control over occupations and enterprises has been exercised by district associations which tend to be formed on the basis of the district in Kwangtung Province from which most of the immigrants came. Sze Yap peoples, from the southern-most districts, controlled many laundries, restaurants and shops, while the Sam Yep people from the districts close to Canton, once did most of the tailoring and were herbalists and merchants. Hakkas, who arrived in Kwangtung Province from the north several centuries ago, and are thought to be tribally distinct, were seamen and cooks, and practised in the United States a type of nepotism in controlling the latter jobs (Lee, 1960). There is a report (Liang, 1964) on possible geographic differences in the risk of nasopharynx cancer within Kwangtung Province, and this inhibits a simple interpretation of the economic difference.

### INTERPRETATION

*An environmental hypothesis*

Because the incidence of nasopharynx cancer is unusually high not only in the Chinese, but also in the Malays, a Mongoloid people that have had long contact with the Chinese, Marsden (1958) has expressed the idea that something more than an external carcinogen must be involved. While the reduced incidence in the U.S. born Chinese does suggest an external carcinogen, there are theoretical reasons why Marsden's proposal should not be dismissed. One of these is the difficulty in conceptualizing an appropriate environmental hypothesis.

The suggestions that have been made for an environmental carcinogen to explain the Chinese risk, unventilated smoky rooms and the burning of incense, run against the objection that Chinese women should have the greatest exposure. Yet their rates are only about one-third of the rates for males. Another way to approach the problem is to ask how an external carcinogen would operate on the Chinese population to produce the rates they experience. It is unlikely to be explained only by repeated exposure to small doses over a long period of time, as in the form of inhalation of particulate matter, for the following reason.

Doll (1963) points out that most of the epithelial cancers of the respiratory, digestive and urinary tract characteristically display a continuous and rapid rate of increase of incidence between ages 30 and 70. The logarithm of rates plotted against logarithm of age is linear and rates increase approximately in proportion to the sixth power of age, i.e., when age is doubled, incidence increases about 64

FIG. 2.—Average annual cancer death rates on a log-log scale.

times. Doll was able to get the same proportionate increase of mortality with age when plotting the lung cancer rates for British doctors who continued to smoke cigarettes. He suggests this might be taken as representative, and that similar rapid and continuous increases of rates may be interpreted as the " result of repeated exposure to carcinogenic stimuli of approximately equal strength ". But the relationship of age and nasopharynx cancer rates is quite different.

In Fig. 2 both age and mortality rates from nasopharynx cancer are shown on logarithmic scale, along with the rates for lung cancer. There is a linear relationship up to about age 70 for lung cancer in Caucasoid males and possibly in Chinese males, the rates increasing in proportion to about the seventh power of age, slightly higher than others have found. It is uncertain that the Chinese nasopharynx cancer rates follow such a relationship, even up to age 55, any better than they followed an exponential relationship in Fig. 1, and that was doubtful. But the point is that even if they do form a logarithmic relationship, the slope of the line is much less steep than that for lung cancer ; the rates increase in proportion to less than the second power of age, i.e., the slope is less than 2 : 1. For the Caucasoid males, also in Fig. 2, the slope is about 3 : 1.

I do not believe the data permit a decision about the relationship between age and the nasopharynx cancer rates, especially with the latter expressed in 10-year age groups. I am suggesting, however, that nasopharynx cancer in the Chinese departs so much from the lung cancer model that the assumption of repetitive carcinogenic stimuli of approximately equal strength must be viewed critically. For nasopharynx cancer is clinically expressed quite early in life (there is more cancer of the nasopharynx than of the lung in Chinese up to age 45) ; therefore, exposure to the repetitive stimuli must start early and, to produce the much shallower slope, cease early or be reduced in amount. The alternative is that exposure stays at the same level but that with maturity the nasopharynx tissues become less susceptible. It is difficult to conceive of an external carcinogen in the form of inhaled particulate matter that starts early, acts repetitively in approximately equal amounts, then ceases early ; so that a change in susceptibility in the Chinese would seem a more likely explanation of the shallow slope—if there is reason to assume repetitive carcinogenic stimuli.

*The reduced risk in the locally born*

There are two things to note about the reduction in risk for the locally born Chinese. First, even with the reduction, the incidence in both men and women is about 20 times greater than that in the Caucasoid. Second, the reduction seems to be proportionately about the same in men and women.

Could the reduction be due to some bias in the data? It could if the enumerated population of locally born Chinese were spuriously inflated, perhaps because immigrants who entered illegally feared for their status, while nativity on the death certificate was correctly reported. But there are several kinds of indirect evidence that this could not be an important bias.

First, one suspects this hypothetical bias to be more prominent among the men than among women, because the latter are less likely to enter illegally. But this should produce a greater reduction of incidence in the men, which did not occur. Second, the number of Chinese persons claiming native birth in the U.S. Census of 1960 compares not too unfavorably with the theoretical number expected to survive out of a birth cohort based on registered Chinese births in the U.S. plus Hawaii during 1921–26 (by which time all States with more than a few dozen Chinese were in the Birth Registration Area). It is about 7 per cent in excess for both male and female, and, interestingly, for white persons.

The destruction of records in the fire following the San Francisco earthquake of 1906 gave an opportunity to claim local birth which could not easily be disproved. This potential source of bias, however, could not affect the difference in incidence between immigrant and locally born under age 50 or so ; and this difference, though not shown in Table II, is proportionately as great as the total difference.

That there was a great deal of illegal entry is not to be doubted. It was unlikely however, to lead to a claim of native birth because of the unique system of illegal entry which developed. This means of entry, known as the " slot racket ", was made possible by a section (1993) of the citizenship law which stated that children " whose fathers were or may have been at the time of their birth citizens, are American citizens too, provided their fathers resided in America at one time " (Lee, 1960). Many of the sojourners periodically visited their wives in

China and their children were born there. Before returning to the United States they would register these births with American Consultates in China or with immigration authorities here. If they had remained long enough they could also report more births than had occurred, or register daughters as sons. This created " slots " which, after a number of years, could be sold to young males, who then claimed immigration rights and derivative citizenship. The practice even gave rise to brokers who made arrangements. Under the slot racket, kinship was falsified rather than birthplace, and, indeed, " proof " of one's legal status as a derivative citizen would depend on a record of China birth. For this and the other reasons mentioned it is unlikely that a bias in reporting nativity can account for the reduced incidence of nasopharynx cancer in the locally born.

*A genetic hypothesis*

A search of the literature found no report of family studies of nasopharynx cancer to test a genetic hypothesis. Pang (1959) did find two pairs of related cases, mother–son and sister–brother combinations, in a series of 34 consecutive nasopharynx cancer cases in Hawaii, including 27 Chinese. And one mother–son pair was found in the 80 California death certificates used in this study Such records are not appropriate for family studies and Pang did not regard his finding as significant, though one might regard it as suggestive.

While the reduced incidence among the U.S. born Chinese would support an environmental hypothesis, it does not in itself rule out genetic etiology. This is true not only because, as Zippen *et al.* (1962) pointed out, one can postulate genetic-environmental interaction. One can also postulate selection against a genotype as a cause of reduced incidence in the filial generation.

Under certain conditions, selection against a genotype is effective enough to reduce the frequency of a trait to a noticeable degree in one generation. Before reviewing these conditions it should be noted that reduction of the probability of death from nasopharynx cancer in the U.S. born generation is about 45 per cent. (Crude estimates of the lifetime probability are 1·1 per cent in foreign born Chinese males, 0·6 per cent in U.S. born Chinese males, and 0·3 per cent in all Chinese women.)

*Recessive inheritance.*—Selection against a recessive genotype in one generation, due to failure to reproduce, is usually quite minimal because the frequency of heterozygotes, i.e., those who carry one of the alleles, is high relative to the homozygotes, those who carry both alleles and express the trait (Stern, 1960). One exception is the sex-linked recessive, but this seems unlikely in the present case because the male/female ratio is of the wrong order of magnitude, being $0{\cdot}011/0{\cdot}003$ rather than $0{\cdot}011/(0{\cdot}011)^2$. But another exception is when the alleles tend to be concentrated in isolates, i.e., mating units or subpopulations. Under these circumstances, more of the alleles are found in the homozygous combination ; selection is then more efficient since in eliminating homozygotes a larger proportion of the alleles in the population is eliminated. A similar argument holds in the case of polygenic rather than single gene inheritance ; a high concentration of polygenic combinations in an isolate will make more individuals subject to selection (Stern, 1960).

*Dominant inheritance.*—Compared to the recessive, selection against a dominant trait, due to the failure of those with the trait to reproduce, is highly efficient.

Under the simplifying assumption of complete penetrance, all carriers express the trait, so that selection is efficient even if the gene for the dominant is diffused throughout the population (Stern, 1960).

*Selection through failure to reproduce.*—Assume, for the purpose of argument, that nasopharynx cancer, or a precursor state, is under genetic control, specifically of an autosomal gene with dominant expression. The immigrant generation arrives with a certain frequency of the allele. Postponement of marriage, together with an early death from nasopharynx cancer, lowers the frequency in the reproducing portion of the population. Added to this, postponement of reproduction of those who were separated from their wives, coupled with early death from nasopharynx cancer, lowers the fertility of the remaining genotypes. And the first generation of locally born then carries a lower frequency of the allele.

There is no doubt that the immigrant generation had a lower fertility than their fathers. Even as late as 1950 the ratio of single adult men to single women was 2 to 1 and several decades earlier the ratio was several times higher. Evidence that marriage was often postponed is revealed by the disparity in parental age on some birth certificates of Chinese, with a paternal age of 40 or 50 and a maternal age of 20 or 30 not infrequent. About 28 per cent of the nasopharynx cancer cases were reported to have died unmarried. And some married men were separated from their wives for long intervals, partly due to the Exclusion Acts of 1882 and 1924 (Lee, 1960).

Death from nasopharynx cancer occurred early enough in many cases to overlap the postponed reproductive age. Some 7 per cent of the deaths occurred before age 30, 28 per cent before age 40, and 45 per cent before age 50.

The potentiality for selection against a dominant genotype is obvious. This does not, however, exclude the possibility of a recessive genotype, or of a polygenic combination giving rise to the trait. But an additional assumption, as discussed earlier, that of an isolate with a high concentration of the recessive alleles or of the polygenic combinations, would be necessary to account for the marked reduction in the incidence of the disease.

The assumption of an isolate may be a tenable one. According to recent reports from China there is considerable variation in the frequency of nasopharynx cancer relative to all cancer cases. The frequency increases from north to south China, being highest in Kwangtung Province (Hu and Yang, 1959 ; Liang, 1964). Even within Kwangtung Province the prevalence is reported to vary as much as 4- or 5-fold from one locality to another (Liang, 1964). (Determinations were made by mass survey using " crude clinical examinations " of 440,000 people over age 20 ; comparability of survey from district to district is not reported.)

If the immigrants to California included individuals from an isolate in which alleles were concentrated in the homozygous state, the potentiality for selection against a recessive genotype must also be recognized. And similarly for polygenic rather than single gene inheritance. Consequently, one cannot select between alternative hypotheses of dominance or recessivity, or between single gene or polygenic inheritance. All that can be recognized is that a potential existed for genetic selection, and this is a possible explanation of the reduction of incidence of nasopharynx cancer in the locally born Chinese.

The development of a genetic hypothesis for nasopharynx cancer must take account of a 3 to 1 male/female ratio and this is enough to show that the hypothesis could not be simple. Among the possibilities are a single gene effect in a geneticenvironmental interaction, to account for sex control of expression ; or polygenic traits forming a threshold on the basis of which sex-modification of the trait takes place. A good example of the latter is Carter's (1961) model, for congenital pyloric stenosis, of a genotype having 2 components, a common dominant gene, and a sex modified multifactorial background.

### DISCUSSION

The reduced incidence of nasopharynx cancer in the locally born Chinese does not provide a clear cut decision between an environmental and a genetic hypothesis. Nor does it make an environmental hypothesis any more persuasive as long as it is reasonable to entertain the hypothesis of selection against a genotype.

A simple environmental hypothesis, involving repeated exposure to particulate matter through inhalation, does not seem an attractive hypothesis in the light of the age distribution of rates of nasopharynx cancer. It is true that this hardly exhausts the possibilities ; a virus, or some other encounter with external carcinogens, may, in the final outcome, be true. Indeed, since chronic and allergic rhinitis and sinusitis are said to have been long prevalent in China (Editorial, 1959), traditional methods of treatment may have included a carcinogenic substance. Nevertheless, a genetic hypothesis is an attractive one : partly because the disease is rare outside of Chinese and Malay populations, partly because the risk is carried overseas even to the second generation, but also partly because of the deductions that can be made from it.

For example, where overseas Chinese colonies were established by intact families, no genetic selection, and no reduction of disease incidence would occur. Such colonies would be scarce, perhaps non-existent, though segments of colonies may have such history. Where fertility was low in the early history of a colony, which then establishes a more normal pattern of marriage and age at reproduction, as in California, incidence rates will reduce no further. Finally, family studies would test the hypothesis, though they might be carried out in difficult circumstances among the Chinese whose immigration status was clouded.

### SUMMARY

Chinese who were born in the United States have about 20-fold greater mortality from nasopharynx cancer than the Caucasoid population of California. Immigrants from China have 30- to 40-fold greater mortality. An external carcinogen cannot, yet, be inferred from the reduced risk in the locally born because the reduction might be caused by selection against a genotype. While the evidence shown does not prove genetic inheritance, it does show that conditions for selection were present.

### REFERENCES

CARTER, C. O.—(1961) *Brit. med. Bull.*, **17**, 251.
DIGBY, K. H., FOOK, W. L. AND CHE, Y. T.—(1941) *Brit. J. Surg.*, **28**; 517.
DJOJOPRANOTO, M. AND MARCHETTA, F. C.—(1959) *Arch. Otolaryng., Chicago,* **69**, 155.
DOLL, R.—(1963) *Cancer Res.*, **23**, 1613.
EDITORIAL—(1959) *Chin. med. J.*, **79**, 412.
HU, CHENG-HSIANG AND YANG, CHIEN—(1959) *Chin. med. J.*, **79**, 409.
LEE, R. H.—(1960) ' The Chinese in the U.S. of America ', Hong Kong (Hong Kong University Press).

LIANG, PO-CHIANG—(1964) *Chin. med. J.*, **83**, 373.
MARSDEN, A. T. H.—(1958) *Brit. J. Cancer*, **12**, 161.
MARTIN, H. AND QUAN, S.—(1951) *Ann. Otol. etc., St. Louis*, **60**, 168.
MUIR, C. S.—(1962) *Brit. J. Cancer*, **16**, 583.
PANG, L. Q.—(1959) *Ann. Otol. Rhinol. Lar.*, **68**, 356.
STERN, C.—(1960) ' Principles of Human Genetics ', San Francisco and London (W. H. Freeman).
STOCKS, P.—(1959) *Bull. Wld Hlth Org.*, **20**, 697.
YEH, S. AND COWDRY, E. V.—(1954) *Cancer*, **7**, 425.
ZIPPEN, C., TEKAWA, I. S., BRAGG, K. U., WATSON, D. A., AND LINDEN, G.—(1962) *J. nat. Cancer Inst.*, **29**, 483.


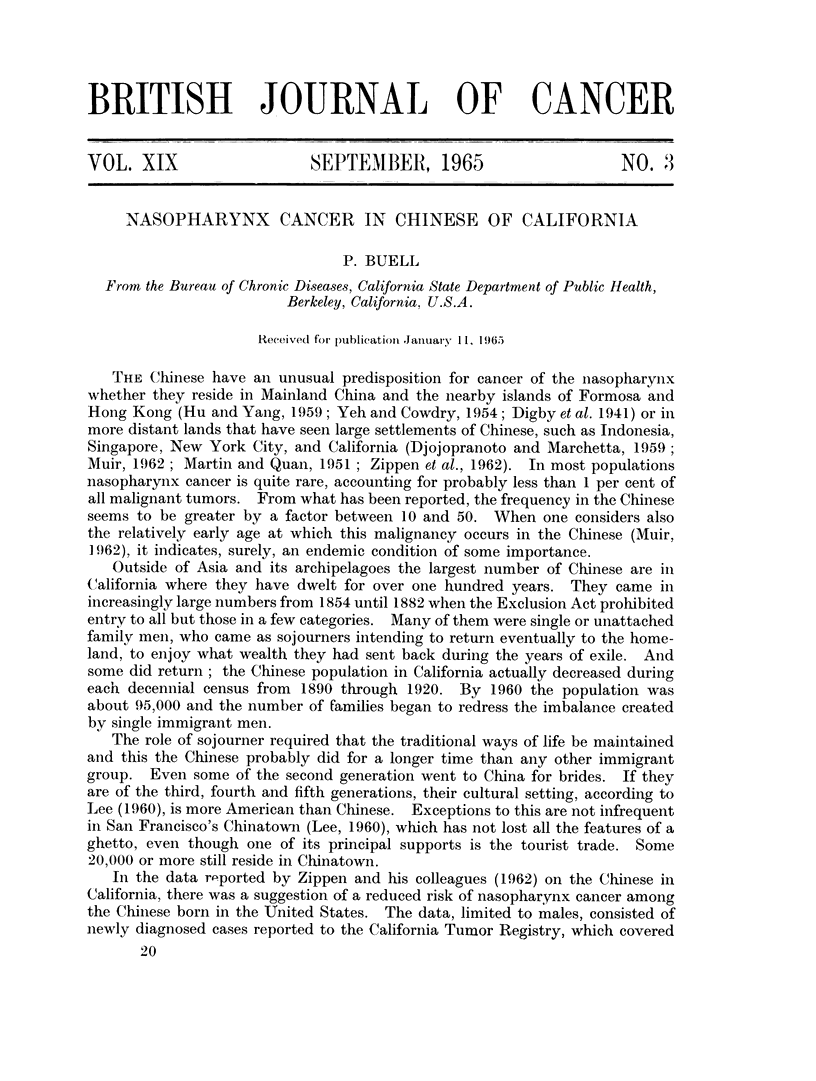

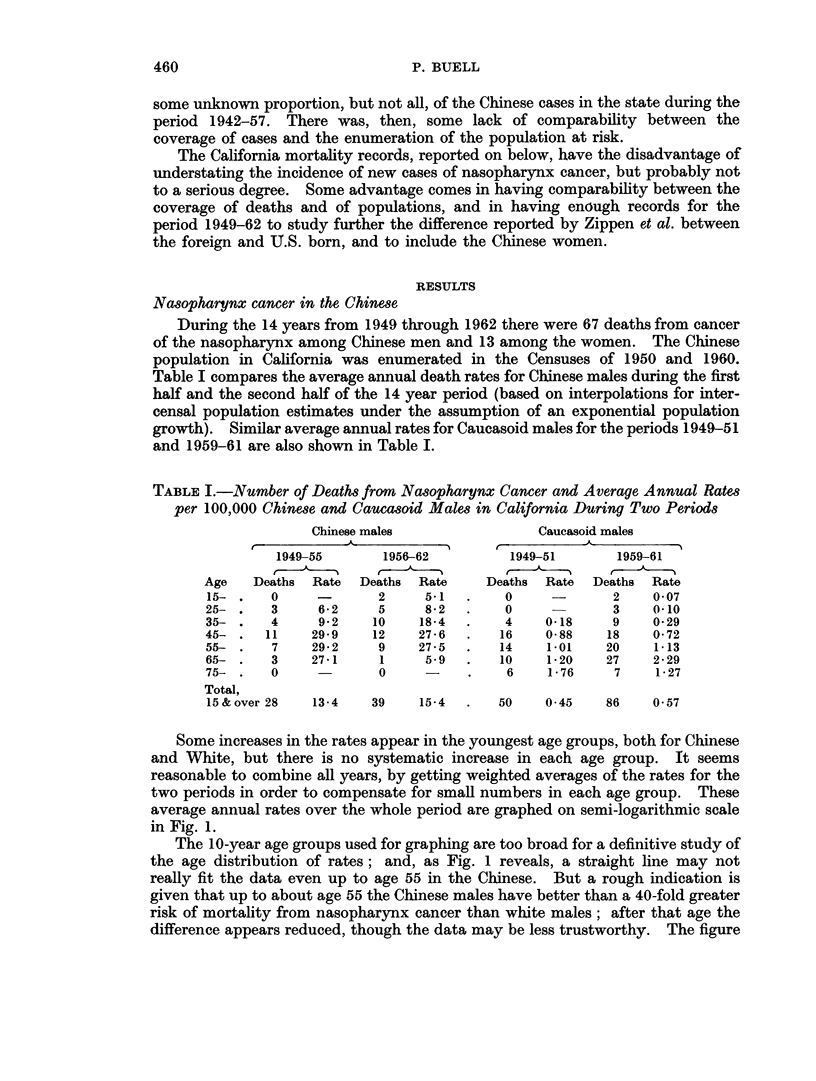

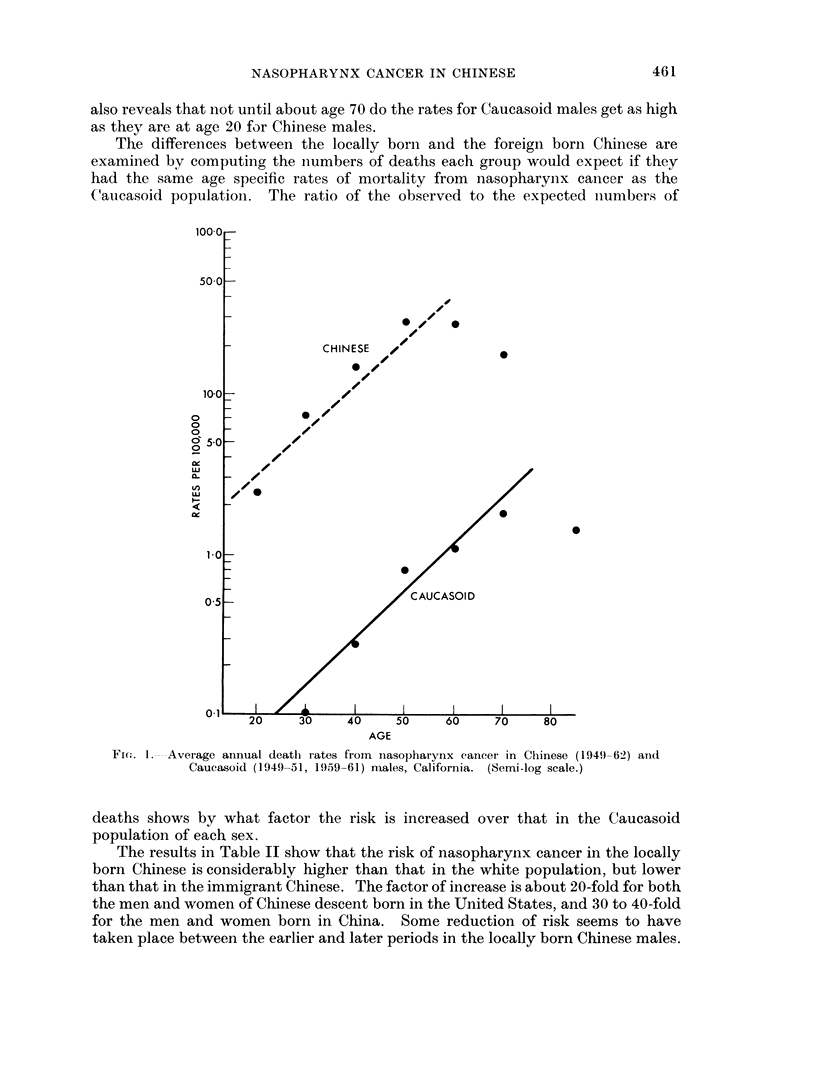

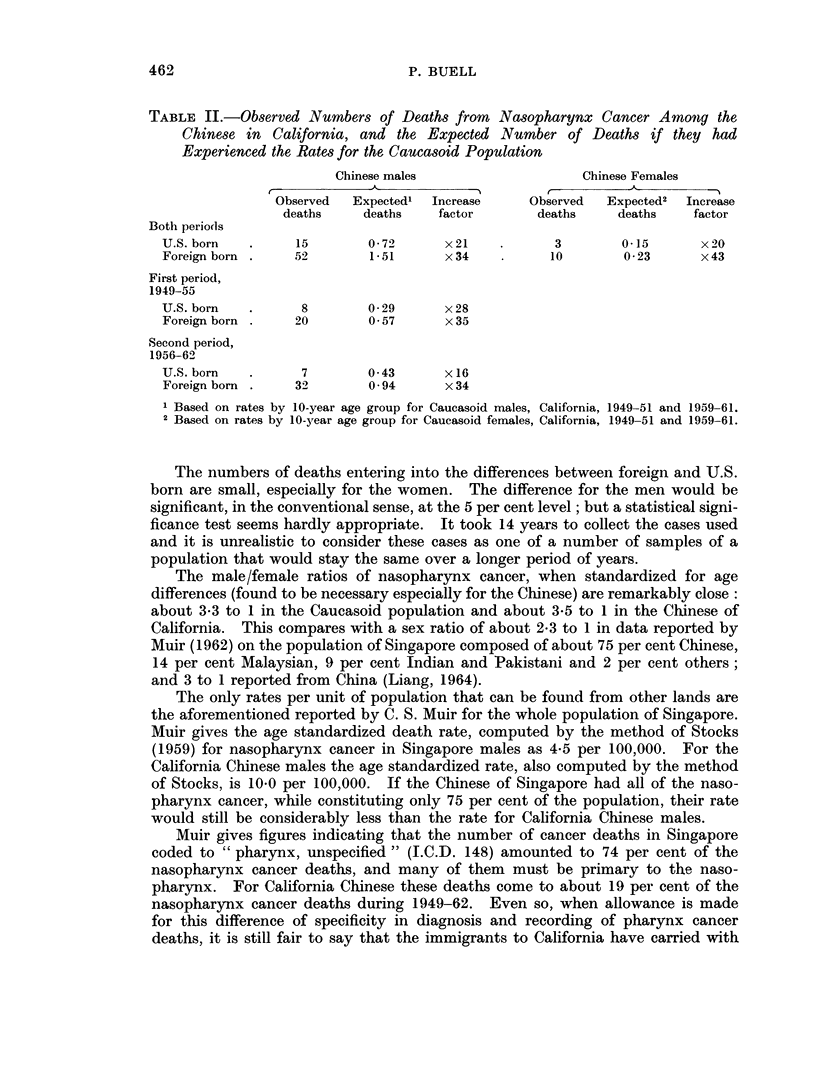

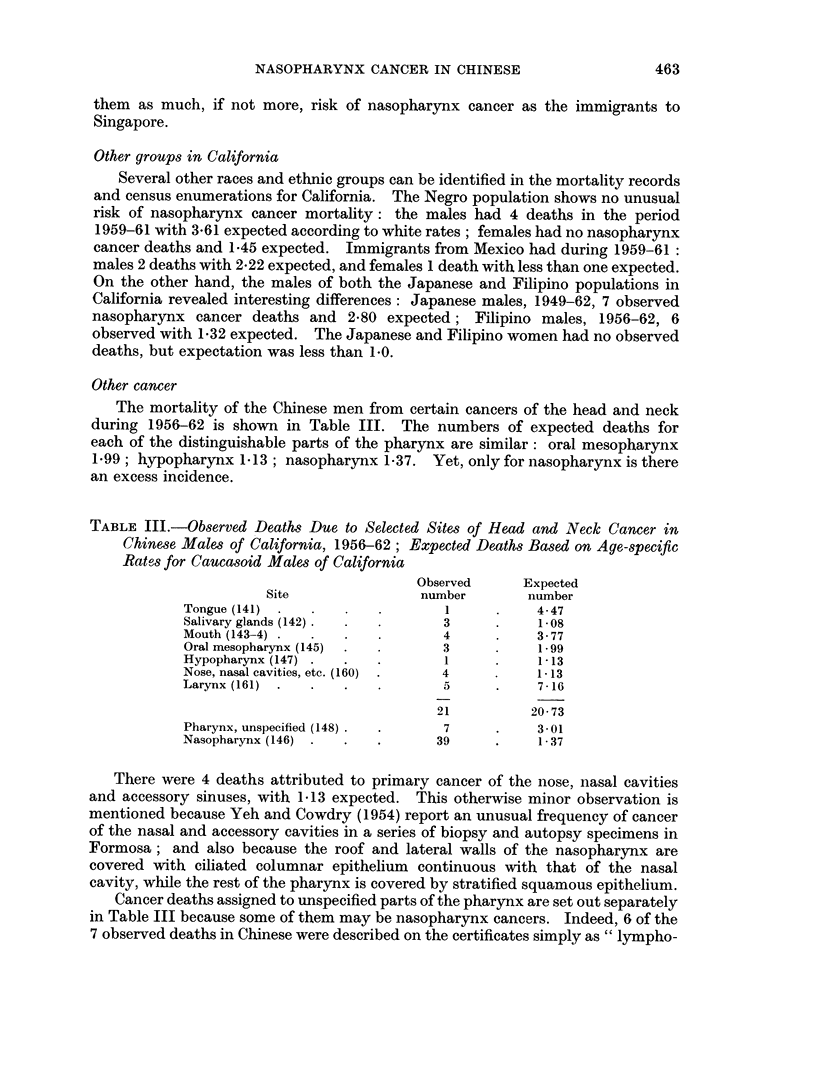

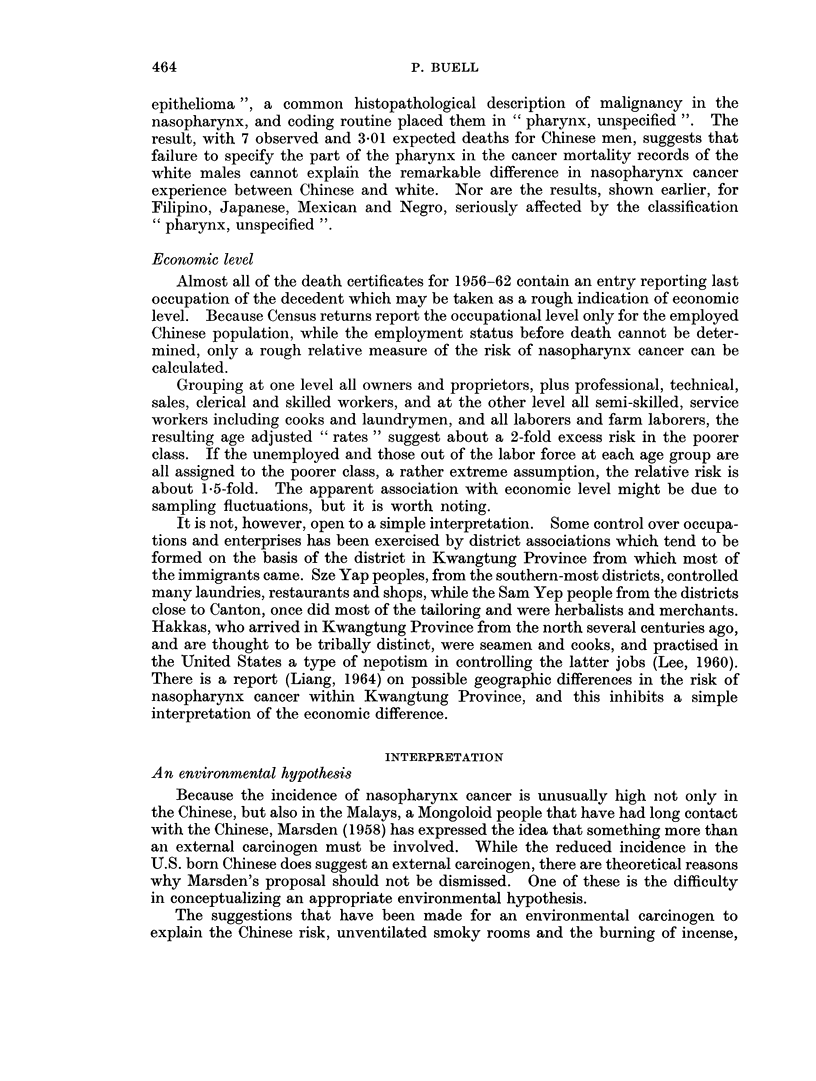

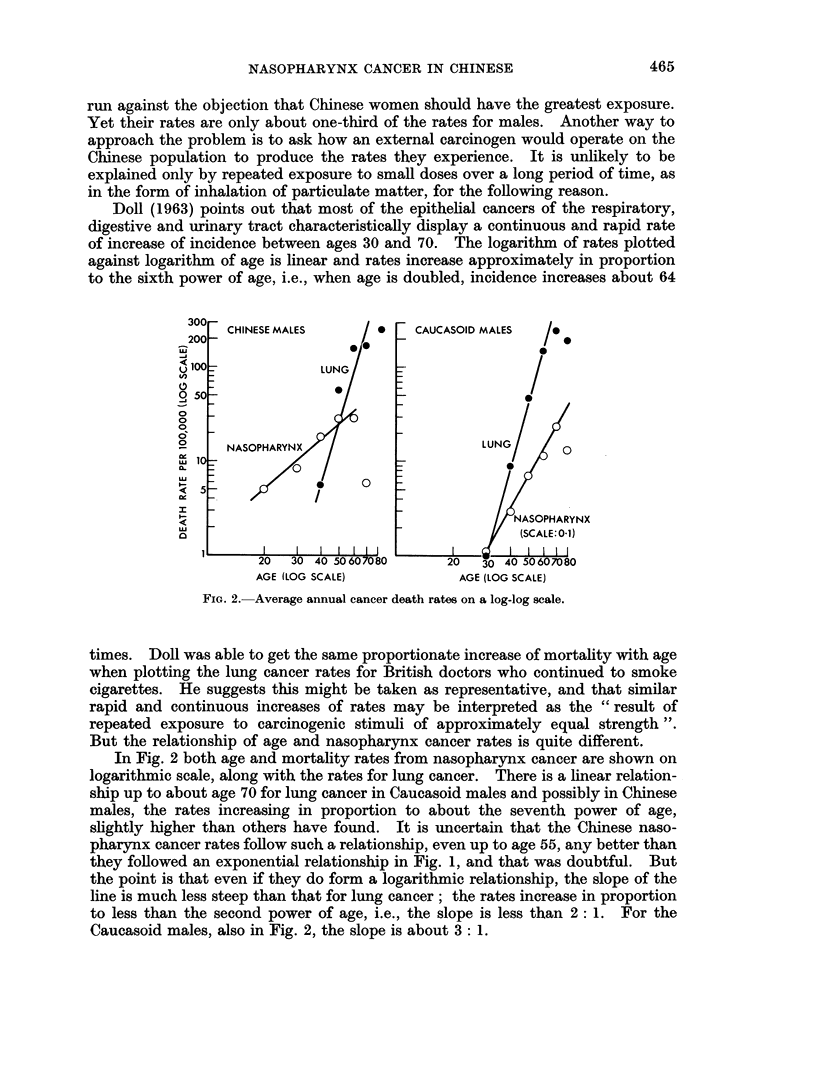

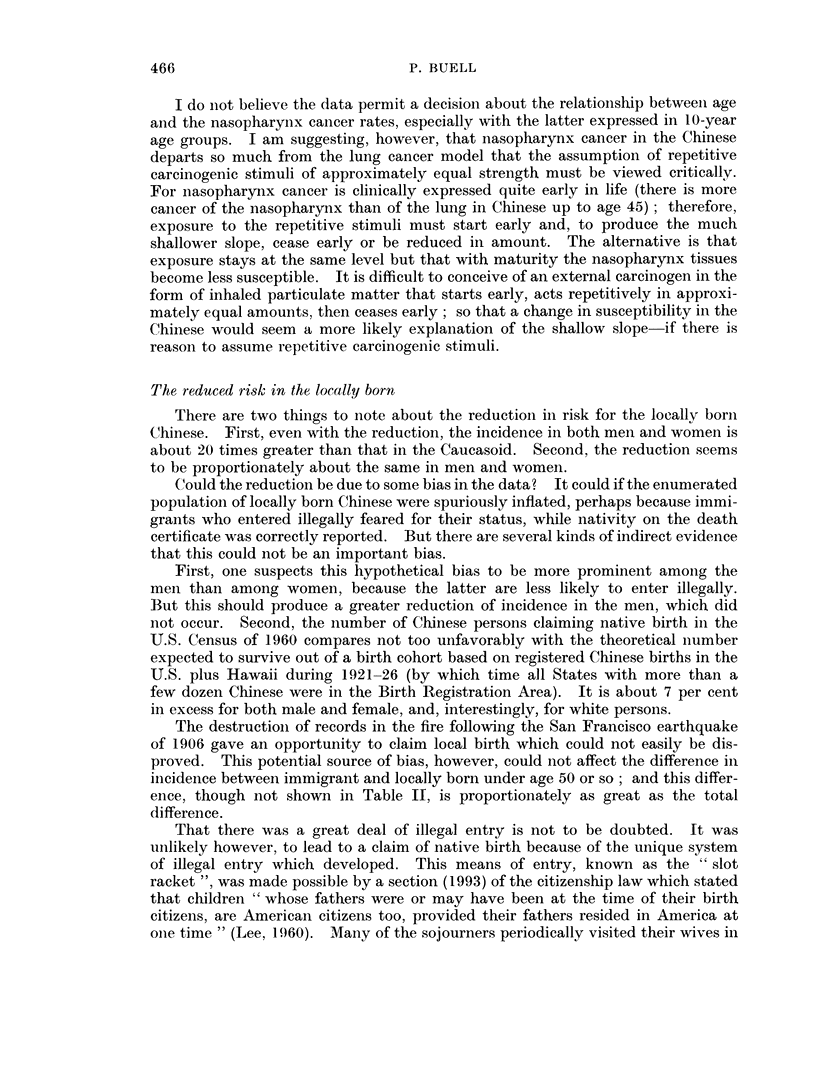

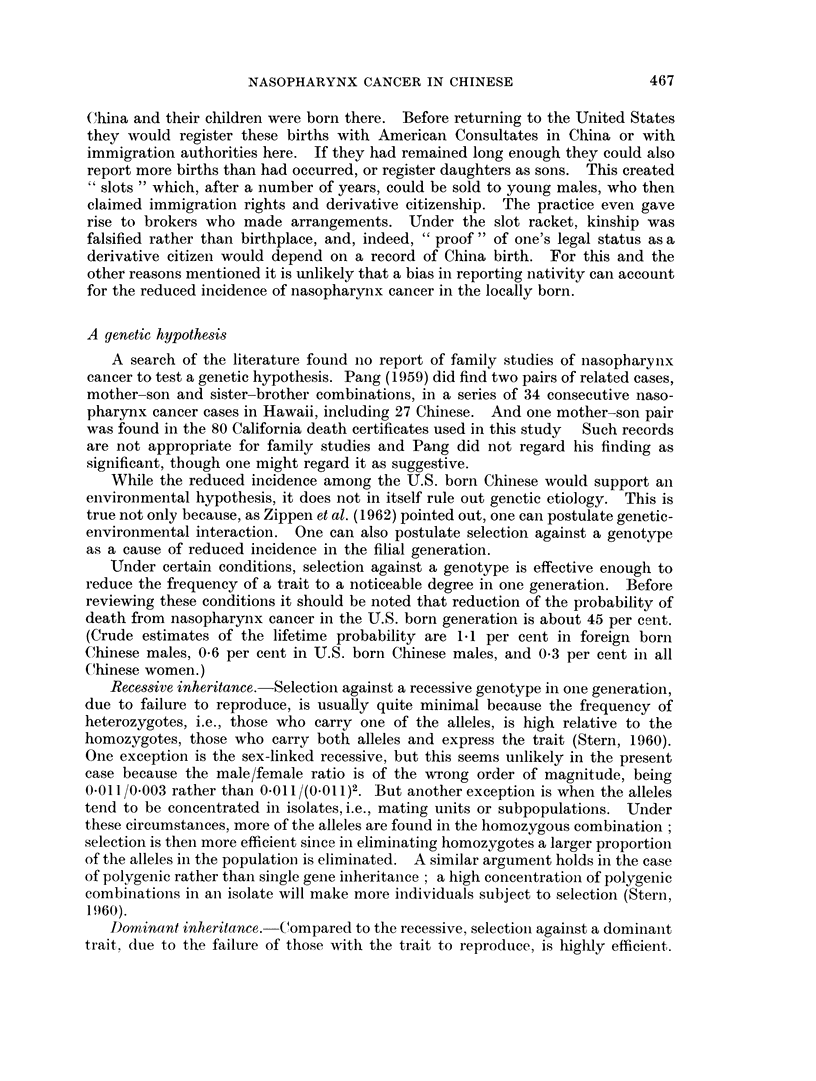

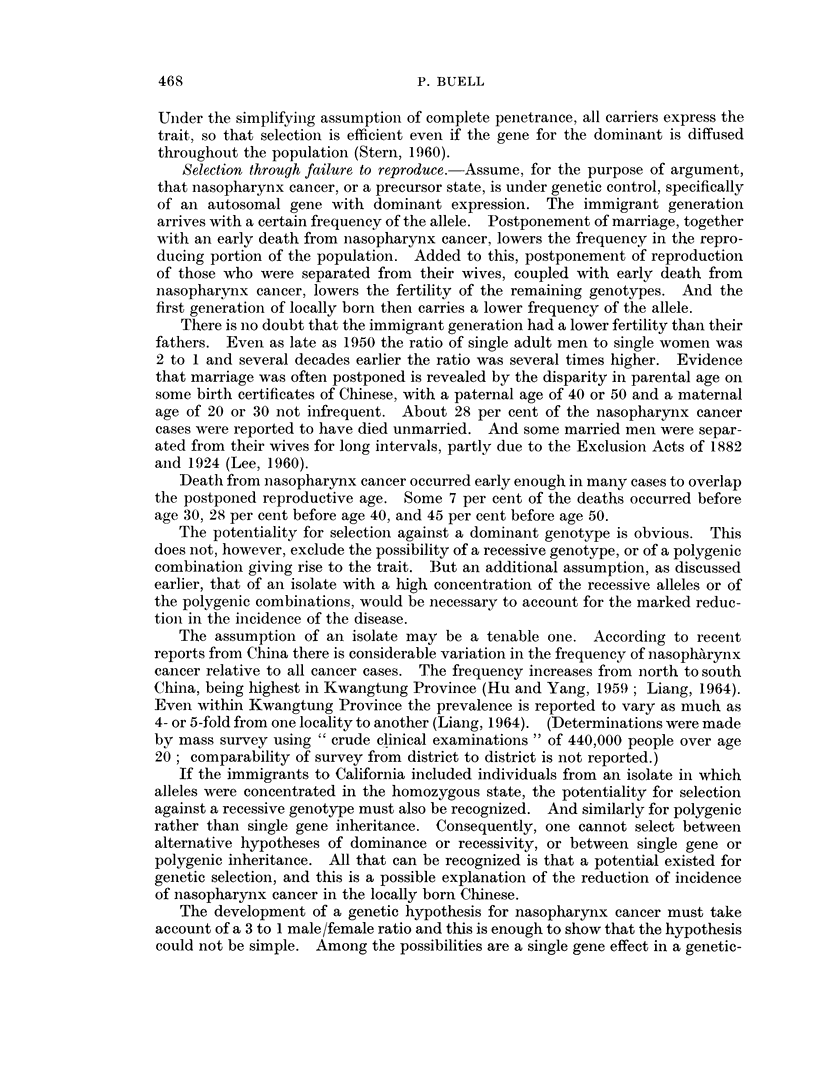

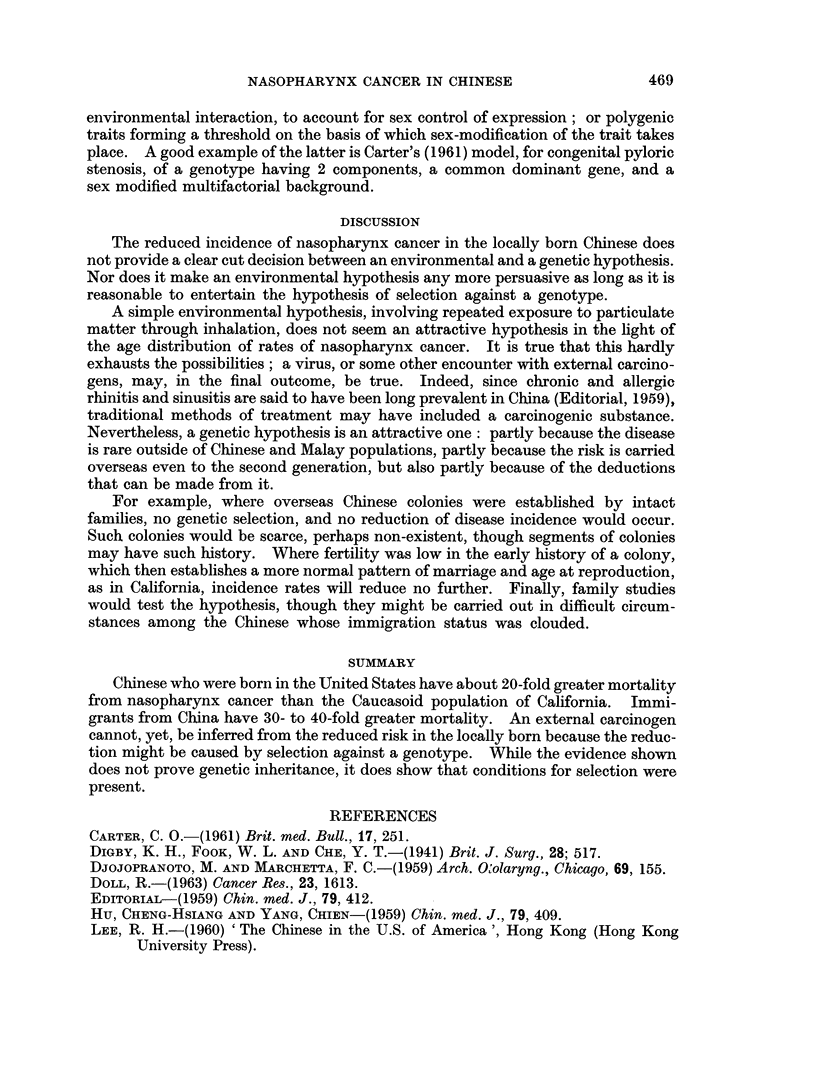

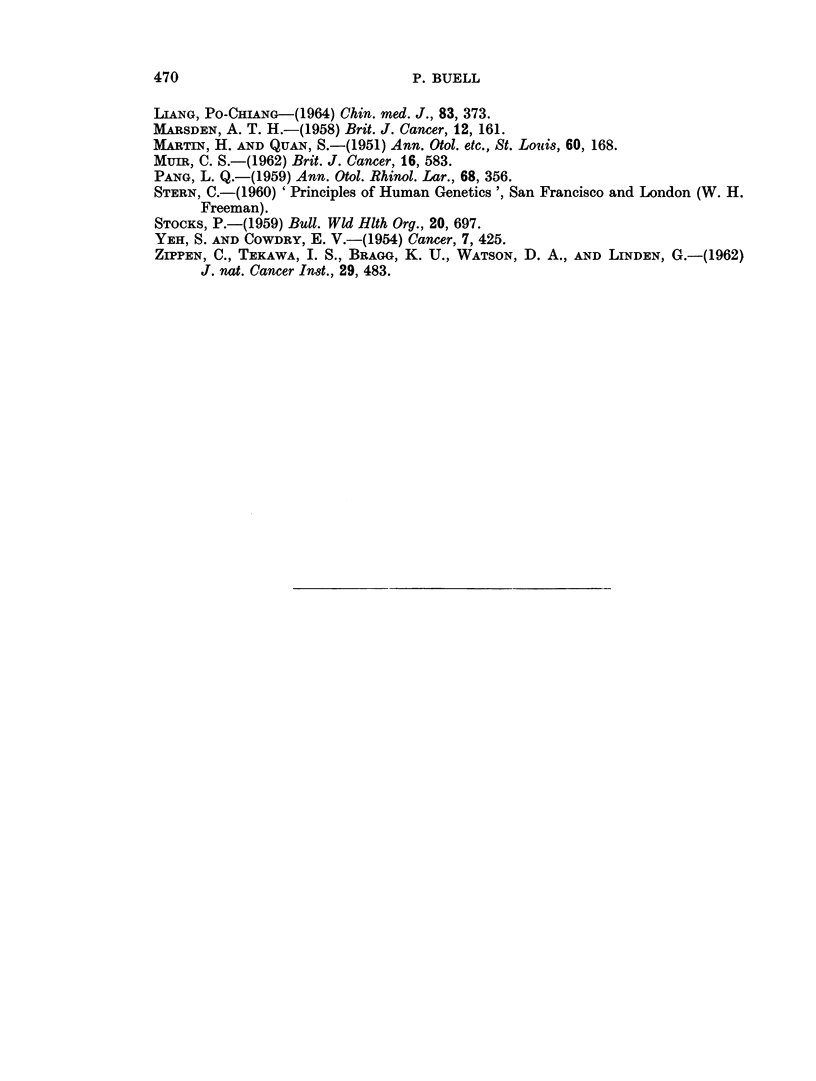


## References

[OCR_00660] DOLL R. (1963). INTERPRETATIONS OF EPIDEMIOLOGIC DATA.. Cancer Res.

[OCR_00666] HU C. H., YANG C. (1959). A decade of progress in morphologic pathology.. Chin Med J.

[OCR_00677] MARTIN H., QUAN S. (1951). The racial incidence (Chinese) of nasopharyngeal cancer.. Ann Otol Rhinol Laryngol.

[OCR_00685] Mackie I. J., Bull H., Brozovic M. (1980). The properties of plasma antithrombin III before and after freezing at -20 degrees C.. Thromb Res.

[OCR_00679] PANG L. Q. (1959). Carcinoma of the nasopharynx; an analysis of thirty-four cases and a preliminary report on palatal fenestration in its management.. Ann Otol Rhinol Laryngol.

[OCR_00687] YEH S., COWDRY E. V. (1954). Incidence of malignant tumors in Chinese, especially in Formosa.. Cancer.

